# The Development of Global-Level Categorization: Frequency Tagging EEG Responses

**DOI:** 10.3390/brainsci14060541

**Published:** 2024-05-24

**Authors:** Stefanie Peykarjou, Stefanie Hoehl, Sabina Pauen

**Affiliations:** 1 Department of Psychology, Heidelberg University, 69117 Heidelberg, Germany; stefanie.peykarjou@psychologie.uni-heidelberg.de; 2 Department of Developmental and Educational Psychology, Charlotte Fresenius University Wiesbaden, 65185 Wiesbaden, Germany; 3 Department of Developmental and Educational Psychology, University of Vienna, 1010 Vienna, Austria; stefanie.hoehl@univie.ac.at

**Keywords:** categorization, global, EEG, frequency tagging, Fast Periodic Visual Stimulation (FPVS)

## Abstract

Adults and infants form abstract categories of visual objects, but little is known about the development of global categorization. This study aims to characterize the development of very fast global categorization (living and non-living objects) and to determine whether and how low-level stimulus characteristics contribute to this response. Frequency tagging was used to characterize the development of global-level categorization in *N* = 69 infants (4, 7, 11 months), *N* = 22 children (5–6 years old), and *N* = 20 young adults. Images were presented in an oddball paradigm, with a category change at every fifth position (AAAABAAAABA…). Strong and significant high-level categorization was observed in all age groups, with reduced responses for phase-scrambled control sequences (R^2^ = 0.34–0.73). No differences between the categorization of living and non-living targets were observed. These data demonstrate high-level visual categorization as living and non-living from four months to adulthood, providing converging evidence that humans are highly sensitive to broad categorical information from infancy onward.

## 1. Introduction

Imagine being a preverbal infant who encounters an object that they have never seen before. It seems most important to decide whether this is a living thing or not, in order to predict whether it will be able to move and act of its own accord. Identifying living entities as such provides the potential evolutionary advantage of treating these more carefully than inanimate objects, independent of the specific type of living or non-living object one encounters. Indeed, under strained circumstances, we can determine whether an image shows a living or non-living object more quickly than identifying which type of object it is [1,2,3], and infants ontogenetically distinguish broad categories prior to more fine-grained ones [4,5].

Traditionally, the categorization hierarchy is assumed to include a basic level, differentiating everyday object classes such as cats, dogs, cars, and motorcycles at a superordinate level in which these are summarized (e.g., mammals, vehicles), and a subordinate level differentiating category membership in more detail (e.g., Maine Coon or Siamese, Labrador or Beagle). Developmental research has expanded this hierarchy by including an even broader level spanning the living/non-living distinction, the so-called global level [6]. Evidence for such global-level categories comes from fMRI studies in adults [7,8] and behavioral studies in children and infants [8,9,10,11]. During infancy, categorization responses develop from broad and inclusive categories to more circumscribed categories, the so-called global-to-basic-level shift [4,11]. Converging evidence for this shift has been found across a variety of paradigms, with slight differences in the developmental timing. While most studies employed gender-balanced designs, no study tested for gender effects on categorization.

Mapping the development of global-level categorization has been aggravated by stark methodological differences between the age groups under study. Research with adults has employed (ultra-)rapid visual categorization, with response times and event-related-potentials (ERPs) as dependent variables, and fMRI for evaluating category-specific neural activation patterns [1,3,7]. In contrast, research on the early development of categorization has mainly relied on behavioral paradigms such as matching-to-sample and object sorting tasks in children [10] or habituation–dishabituation measures and sequential touching in infants [9,11,12]. More recently, ERPs have been employed to evaluate infant categorization, but the living/non-living distinction was not directly tested, and paradigms as well as dependent variables differed from prior work with adults [13,14,15]. It is likely due to these methodological challenges that prior studies have never mapped developmental trajectories over broader periods (e.g., from infants to children, from children to adults).

Recent developments employing frequency tagging in combination with recording EEG provide an opportunity for investigating the development of categorization, as similar methods can be employed across the life-span [16,17]. In this paradigm, changes between categories are embedded into a fixed stimulation sequence and tagged at a specific frequency, defined a priori. EEG responses at this and related frequencies can be employed as an index of categorization in a similar fashion in all age groups. With this approach, it has been demonstrated that infants categorize animal and furniture items from four months onward, but characteristics of adult categorization emerge around eleven months and become more pronounced in preschool children [16]. Adults showed strong categorization responses in occipital-posterior and frontal areas, which were similar for animal and furniture targets. Five-to-six-year-old children showed strong responses in the same areas, and partly enhanced categorization of animal compared to furniture targets. Infants only showed categorization responses in the occipital-posterior region, and an advantage for animal targets emerged by eleven months of age. Phase-scrambled control sequences were employed to evaluate the contribution of low-level visual cues to categorization that are known to be associated with category membership [18]. The categorization responses for adults, children, and eleven-month-olds were much reduced for the phase-scrambled control sequences, indicating categorization based on high-level cues. While there was no statistically significant difference between the responses to the original and phase-scrambled images at four and seven months, significant categorization responses were only observed for original images at these ages.

Thus, high-level visual cues drove categorization responses from eleven months onward, but small yet significant categorization was also evident for stimuli containing only low-level visual cues. Low-level cues refer to local measurements such as orientation, spatial frequency, luminance, and contrast [19], while high-level visual cues refer to the organization of stimulus parts into a global “Gestalt”, the configuration of an entity (cf. APA Dictionary of Psychology, n.d.). Several lines of work have demonstrated that among low-level cues, curvilinear features contained in images of living objects are sufficient to elicit global categorization [20,21], but neural differentiation is maintained when controlling for shape differences associated with category membership [7,22]. Existing studies exploring the role of low-level visual cues have recruited gender-balanced samples but have not yet taken participant gender into account in their statistical analyses.

The current study aims to enhance our understanding of high-level categorization, testing the global-level contrast in infants, children, and adults using EEG frequency tagging. By employing a similar approach across age groups and comparing the categorization of original and phase-scrambled control images, these data provide evidence on the trajectory of high- and low-level categorization. To allow for detecting potential differences compared to a more circumscribed categorical contrast, this study closely followed recently published work on animal–furniture categorization [16]. The youngest age group included was four months, the earliest age at which the categorization of objects as living and non-living has been confirmed across studies [8,23], despite some evidence for earlier categorization [5]. Similar to [16], seven- and eleven-month-olds were included to cover potential developmental changes throughout the first year of life. The general paradigm and all hypotheses are based on this prior work. Strong categorization in the frontal and posterior regions was expected for adults and children, with an advantage for categorizing living targets only in children. In infants, categorization was expected only in the posterior region. Based on the evidence for a global-to-basic-level shift [4,11], categorization as living/non-living was hypothesized to emerge even earlier than categorization as animal/furniture item. It was expected to be stronger for the original than phase-scrambled control sequences from at least seven months of age. In contrast, gender was not expected to have a significant impact on categorization performance at any age because the living/non-living distinction is of high relevance irrespective of sex/gender. Moreover, no previous study reported any relevant gender-related differences regarding categorization responses.

Overall, the hypotheses could be confirmed, with strong categorization responses independent of low-level confounds in adults, children, and even infants starting at four months of age.

## 2. Experiment 1

### 2.1. Materials and Methods

#### 2.1.1. Participants

Twenty adult subjects were tested (13 females, mean age = 21 years, SD = 2 years) after giving written consent to participate. They received course credit for participation. Five additional participants were tested but excluded due to a technical error (missing triggers at the sequence start). Data were collected in Heidelberg, a mid-sized German university town.

#### 2.1.2. Stimuli/Presentation

**Figure 1:**
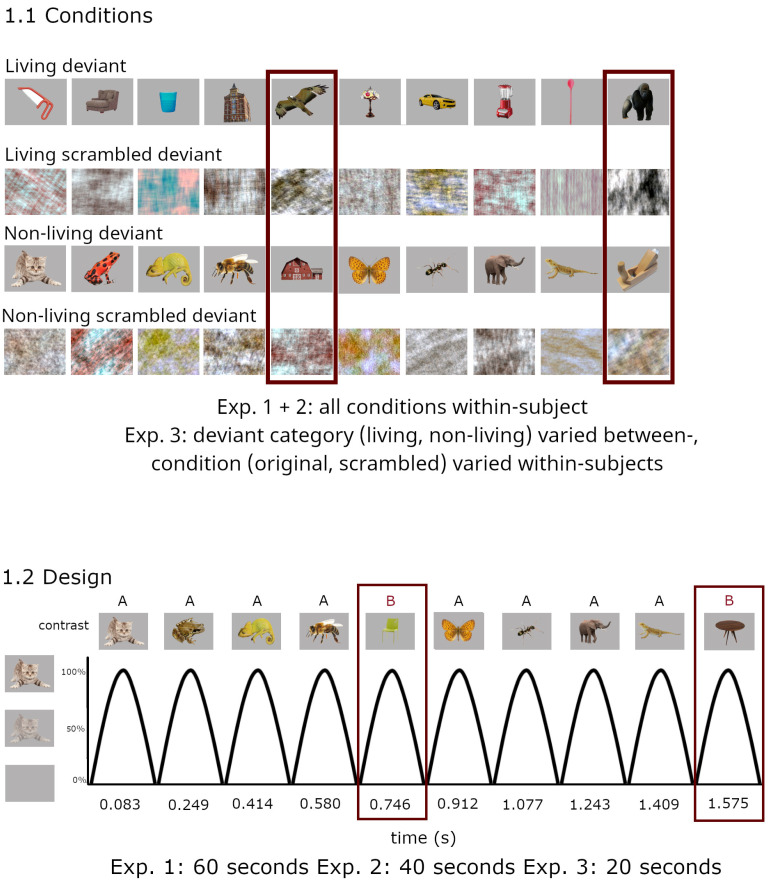
Schematic illustration of the experimental paradigm and conditions. (**1.1**) Four conditions were tested within-subjects in adults and children (Experiments 1 + 2). In infants (Experiment 3), the condition (original, scrambled images) was varied within-, and the deviant category (living, non-living) between-subjects. (**1.2**) Images are presented by sinusoidal contrast modulation at a rate of 6 cycles per second = 6 Hz (1 cycle ≈ 170 ms). Category changes were introduced at fixed intervals of every fifth image (6/5 Hz = 1.2 Hz). Sequence duration was set at 60 s in adults, 40 s in children, and 20 s in infants to accommodate differences in attention span.

Overall, the hypotheses could be confirmed, with strong categorization responses independent of low-level confounds in adults, children, and even infants starting at four months of age. The presentation was similar to recent studies employing frequency tagging [16,24], and full details can be found in Supplementary File S1. Stimulation sequences were displayed at 6 Hz using sinusoidal contrast modulation. At every fifth position, corresponding to 1.2 Hz, the stimulus category changed (6 Hz/5; i.e., AAAABAAAABAAAAB…). EEG amplitude at 1.2 Hz and harmonics (i.e., 2F/5 = 2.4 Hz, 3F/5 = 3.6 Hz…) was used as an index of categorization [17], whereas amplitude at 6 Hz and harmonics was employed to evaluate general attention to the visual display. The schematic stimulation course is illustrated in Figure 1.

Four conditions were presented: living deviant (non-living standard), non-living deviant (living standard), and the corresponding phase-scrambled versions. Per condition, four 60-s sequences were presented, with the order of sequences randomized across participants. Participants were seated at a looking distance of approx. 60 cm from the computer screen and were instructed to watch the presentation passively, but attentively.

#### 2.1.3. EEG Recordings and Analyses

EEG was measured using a BrainProducts actiCap (Gilching, Germany) with 32 active Ag-AgCl electrodes arranged according to the 10-10-system and a right mastoid reference. Sampling rate was set at 250 Hz and the signal was amplified via a BrainAmp amplifier.

#### 2.1.4. EEG Preprocessing and Frequency Domain Analyses

EEG was preprocessed using Letswave (https://www.letswave.org/, accessed on 1 January 2018) and MATLAB 2012b (The Mathworks, Natick, MA, USA) and followed the procedure described in several recent studies [16,24]. A description and flowchart of all steps can be found in Supplementary File S2.

To measure the strength of activity, baseline corrected amplitudes (bca) were computed by subtracting the average amplitude of the 24 surrounding bins (12 on each side excluding the immediately adjacent bins, and two extreme bins) from every frequency bin [24]. Bca was employed for comparing the conditions statistically. Z-scores were calculated as the difference between the amplitude at the frequency of interest and the mean amplitude of 24 surrounding bins divided by the standard deviation of those bins [25] and served to identify significant responses. Threshold of significance was placed at Z-score 1.64 (*p* < 0.05, one-tailed). Signal-to-noise ratios (SNRs) were computed by dividing the signal by the amplitude at the neighboring frequency bins and employed to visualize the response patterns.

Electrode clusters were based on previous work [16,22] and visually verified using heatmaps for individual harmonics. For the categorization response, the posterior-occipital cluster consisted of 11 electrodes (P3, P4, Pz, P7, P8, PO9, PO10, O1, O2, Oz, Iz), and the frontal cluster of 10 electrodes (F3, F4, Fz, C3, C4, Cz, FC1, FC2, FC5, FC6). For the base response, which was primarily analyzed as a control of visual attention directed to the different conditions, analyses were limited to occipital electrodes (O1, O2, Oz). Comparisons between conditions were performed using summed baseline-corrected amplitudes (bca) across consecutively significant harmonics (see Supplementary File S1 for details). Responses were averaged across electrodes per cluster for analysis. Group analyses were calculated by averaging the individual amplitude spectra, then computing bca, SNR, and Z-scores on the resulting grand-averaged spectrum.

#### 2.1.5. Statistical Analyses

Conditions were compared using the baseline corrected amplitudes in a JZS Bayes factor repeated measurement analysis of variance (rmANOVA) with default prior scales [26,27,28]. The Bayes factor rmANOVA provides a more conservative test than the standard rmANOVA and estimates probability for models based on the null and alternative hypotheses, thereby providing scales for interpreting the strength of evidence for H0 and H1. Preliminary analyses did not favor an effect of gender, as all BFs_10_ < 2.91, so gender was not considered in the main analyses. The within-subjects ANOVAs were composed as condition (2: original, scrambled) * deviant category (2: animal, furniture). Effect sizes were computed as the increase in R^2^ when adding the factor to the null model. Analyses including electrode as a factor can be found in Supplementary File S3, and fully confirm the main analysis.

### 2.2. Results

#### 2.2.1. Categorization Responses

**Figure 2:**
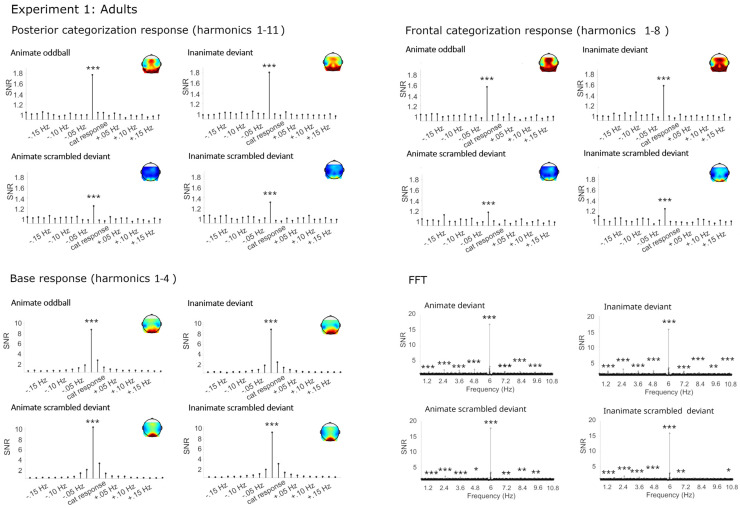
Electroencephalographic responses in Experiment 1 (adults). Signal-to-noise ratios (SNRs) of summed responses for the categorization response at the posterior-occipital and frontal leads and of the base response at the occipital channels. Fast Fourier transformation (FFT) responses at the posterior-occipital channels. Data have been averaged across electrodes per cluster and grand-averaged across participants for display. * = *p* < 0.05, ** = *p* < 0.01, *** = *p* < 0.001.

In the posterior-occipital and the frontal cluster, strong and highly significant categorization responses were obtained for living and non-living deviants (see Figure 2 and Table 1), consistent with rapid categorization in visual and anterior networks. Small but significant categorization responses were also obtained for the phase-scrambled control sequences. This finding reflects neural sensitivity to associations between low-level features with category membership. Consistent with the assumption that Gestalt information and prior knowledge facilitate categorization, responses in the scrambled control conditions were severely reduced compared to the original image sequences.

##### Categorization Responses in Posterior-Occipital Cluster (Harmonics 1–11)

Significant categorization responses were obtained when averaging across channels in all four conditions (all Zs > 5). A main effect of condition provided extreme evidence for a stronger categorization response in the original than phase-scrambled images, BF_10_ = 6.00 × 10^15^, R^2^ = 0.64 (original *M* = 0.42 µV, *SD* = 0.16; scrambled *M* = 0.12 µV, *SD* = 0.08). There was anecdotal evidence against a main effect of category, BF_10_ = 0.43, and against an interaction of category and condition, BF_10_ = 0.43.

##### Categorization Response in Anterior Cluster (Harmonics 1–8)

Significant categorization responses were obtained when averaging across channels in all four conditions (all Zs > 4). There was extreme evidence for stronger responses in the original rather than the phase-scrambled conditions, BF_10_ = 6.61e^9^, R^2^ = 0.71 (original *M* = 0.24, *SD* = 0.09; scrambled *M* = 0.08, *SD* = 0.05). There were no other effects, with evidence speaking against differences between animal and furniture deviants, BF_10_ = 0.26.

#### 2.2.2. Base Frequency (Harmonics 1–4)

Large responses at the base stimulation frequency and its harmonics were observed in all conditions over the medial occipital cortex, with a maximum at O1, O2, Oz. Visual inspection revealed that the amplitude was higher in the phase-scrambled than original conditions. This was verified by the Bayesian ANOVA, BF_10_ = 18,686.70, R^2^ = 0.46.

### 2.3. Summary

Together, these data are consistent with the view that adults quickly and expertly categorize stimuli based on animacy, and that this ability is partly based on low-level cues. To investigate the development of this high-level perceptual categorization, children aged five to six years were tested using the same procedure in Experiment 2.

## 3. Experiment 2

### 3.1. Materials and Methods

The materials, procedure, and statistical analysis were similar to Experiment 1. Only deviations will be detailed in the following paragraphs.

#### 3.1.1. Participants

The final sample consisted of *N* = 22 five-to-six-year-old children (12 females, M age = 75.2 months, SD = 5 months, age range 64–82 months). All children were born full-term (>37 weeks of gestation) and did not report any neurological or visual problems. An additional *N* = 18 children were tested but not included in the final analyses due to a lack of cooperativeness (*N* = 2), bad data (too many artifacts, leading to the exclusion of trials, *N* = 14), or technical errors (*N* = 2). Relatively high dropout-rates were due to employing four conditions within-subjects, requiring participants to remain seated and attentive for a relatively long period of time (approx. 30 min, see Stimuli/Presentation). Moreover, some participants felt uneasy with the procedure. In accordance with the terms provided by the local ethics committee of Heidelberg University that approved the general procedure, verbal consent was obtained from the children, and written informed consent from their caretakers.

#### 3.1.2. Stimulation

Sequence length was set to 40 s to accommodate the children’s need for breaks. Four sequences per condition (16 in total) were presented. During capping, EEG preparation, and after every second FPVS sequence, participants were engaged in a game where they could win stickers to keep them motivated and attentive.

#### 3.1.3. EEG Analysis

On average, the participants watched 14.55 sequences per condition (*SD* = 1.37), and a mean of 13.23 (*SD* = 2.09) was kept for analysis after preprocessing (interpolation and sequence exclusion). There were no differences in the number of trials between the four conditions, all BFs_10_ < 0.62. Baseline corrections were performed using bins 2–8 (excluding two extreme bins) rather than bins 2–12 as in Experiment 1 to keep the frequency range comparable. Statistical analyses including the electrode as a factor can be found in Supplementary File S3 and largely confirmed the analyses on the averaged electrodes per cluster.

Preliminary analyses spoke against gender effects, all BFs_10_ < 0.59, so gender was not considered in the main analysis.

### 3.2. Results

#### 3.2.1. Categorization Responses

**Figure 3:**
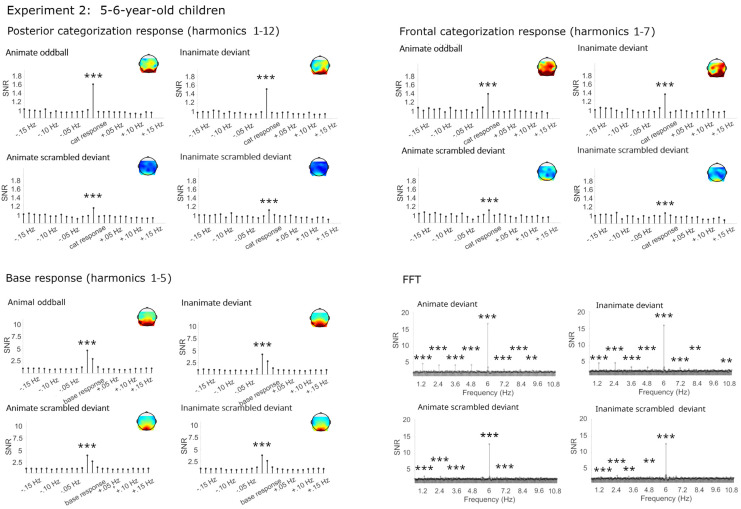
Electroencephalographic responses in Experiment 2 (5–6-year-olds). Signal-to-noise ratios (SNRs) of summed responses for the categorization response at the posterior-occipital and frontal leads and of the base response at the occipital channels. Fast Fourier transformation (FFT) responses at the posterior-occipital channels. Data have been averaged across electrodes per cluster and grand-averaged across participants for display. *** = *p* <0.001, ** = *p* < 0.01.

Similar to Experiment 1, strong and highly significant categorization responses were obtained for living and non-living deviants in both the posterior and the frontal cluster (see Figure 3 and Table 2). Again, small significant but strongly reduced categorization responses were obtained in the phase-scrambled control sequences.

##### Categorization Responses in Posterior-Occipital Cluster (Harmonics 1–12)

Significant categorization responses were obtained when averaging across channels in all four conditions (all Zs > 8). A main effect of condition provided extreme evidence for a stronger categorization response in the original than phase-scrambled images, BF_10_ = 3.75 × 10^14^, R^2^ = 0.73 (original *M* = 1.58 µV, *SD* = 0.58; scrambled *M* = 0.41 µV, *SD* = 0.27). There was anecdotal evidence against a main effect of category, or an interaction of category and condition, BFs_10_ < 0.31.

##### Categorization Response in Anterior Cluster (Harmonics 1–7)

Significant categorization responses were obtained when averaging across channels in all four conditions (all Zs > 3). Consistent with the visual inspection, there was moderate evidence for stronger responses in the original than phase-scrambled conditions, BF_10_ = 9.21, R^2^ = 0.38 (original *M* = 0.70, *SD* = 0.52; scrambled *M* = 0.24, *SD* = 0.48). There were no other effects, with the evidence speaking against differences between animal and furniture deviants, BF_10_ = 0.54.

#### 3.2.2. Base Frequency (Harmonics 1–5)

In all conditions, large responses at the base stimulation frequency and its harmonics were observed over the medial occipital cortex. Visually, there were no differences between conditions, which was verified by the Bayesian ANOVA, all BFs_10_ < 0.34.

### 3.3. Summary

Preschool children showed strong high-level categorization of living and non-living items, similar to the responses of adults recorded in Experiment 1. Next, infants across the first year of life were tested with the same approach to track the development of high-level categorization on the global level.

## 4. Experiment 3

### 4.1. Materials and Methods

Paradigm and analysis were similar to Experiment 1. Only deviations will be detailed.

#### 4.1.1. Participants

The final sample consisted of *N* = 24 four-month-old (7 females, M age = 4 months, 11 days, SD = 10 days), *N* = 24 seven-month-old (11 females, M age = 7 months, 14 days, SD = 9 days), and *N* = 21 eleven-month-old infants (10 females, M age = 11 months, 17 days, SD = 9 days). All children were born full-term (>37 weeks of gestation) and did not report any neurological or visual problems. An additional *N* = 34 children were tested but not included in the final analyses due to bad data (too many artifacts, leading to the exclusion of trials, *N* = 19), fussiness (*N* = 11), or technical errors (*N* = 4). Written informed consent was obtained from the infants’ caretakers.

#### 4.1.2. Stimulation

Sequence length was set to 20 s as customary in infant FPVS studies [15,29], and a mixed design was employed with the condition (original/scrambled) varied within-subjects, and the deviant category (living/non-living) varied between-subjects. A maximum of 16 sequences was presented.

#### 4.1.3. EEG analysis

On average, participants watched 6.43 sequences (*SD* = 1.69) per condition, and a mean of 4.07 (*SD* = 2.06) was kept for analysis after preprocessing (interpolation and sequence exclusion). There was an effect of age group on the number of presented sequences (BF_10_ = 3.30), reflecting a higher number of sequences presented at 7 months of age (4 months *M* = 5.79, *SD* = 1.83, 7 months *M* = 7.14, *SD* = 1.00, 11 months *M* = 6.21, *SD* = 1.86). However, no difference in sequences retained after preprocessing was observed, BF_10_ = 0.58. No effects of condition or category were observed for the numbers of presented or retained sequences, all BFs_10_ < 0.51. Sequences with more than three bad channels were discarded. Baseline corrections were performed using bins 2–6 (excluding no extreme bins) rather than bins 2–12 as in Experiment 1 to keep the frequency range comparable (±0.2 Hz in Experiments 1 + 2, ±0.25 Hz in Experiment 3) while avoiding narrowing down the range of bins too much. Analyses averaged across electrodes per cluster are reported. Analyses including electrode as a factor can be found in Supplementary File S3. Preliminary analyses provided evidence against gender effects, all BFs_10_ < 0.83, so gender was not included in the main statistical analysis.

### 4.2. Results

#### 4.2.1. Categorization Responses

**Figure 4:**
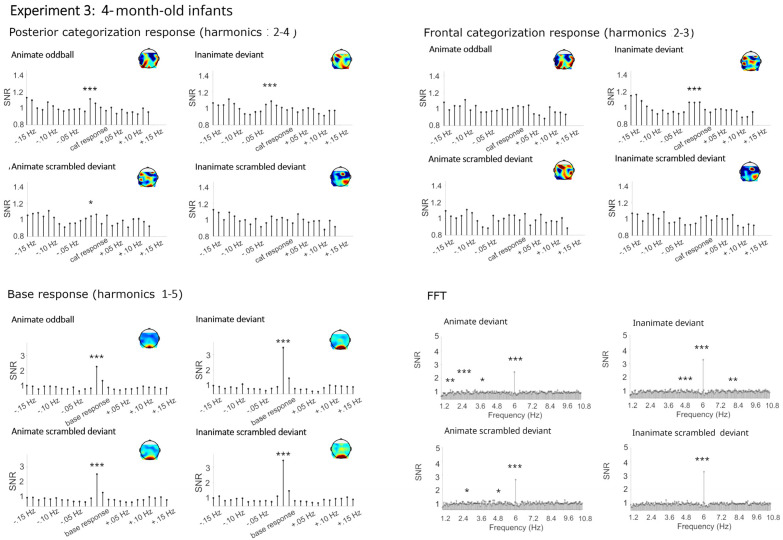
Electroencephalographic responses in Experiment 3 (4-month-old infants). Signal-to- noise ratios (SNRs) of summed responses for the categorization response at the posterior-occipital and frontal leads and of the base response at the occipital channels. Fast Fourier transformation (FFT) responses at the posterior-occipital channels. Data have been averaged across electrodes per cluster and grand-averaged across participants for display. *** = *p* <0.001, ** = *p* < 0.01, * = *p* < 0.05.

**Figure 5:**
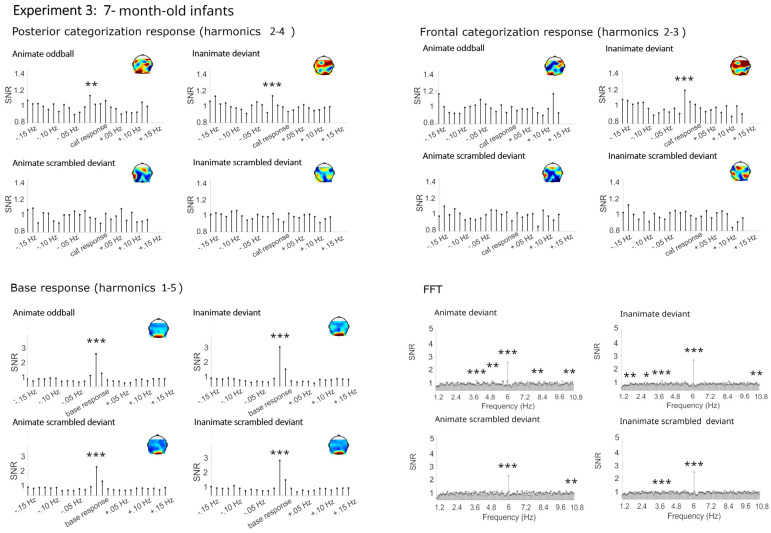
Electroencephalographic responses in Experiment 3 (7-month-old infants). Signal-to-noise ratios (SNRs) of summed responses for the categorization response at the posterior-occipital and frontal leads and of base response at the occipital channels. Fast Fourier transformation (FFT) responses at the posterior-occipital channels. Data have been averaged across electrodes per cluster and grand-averaged across participants for display. *** = *p* <0.001, ** = *p* < 0.01, * = *p* < 0.05.

**Figure 6:**
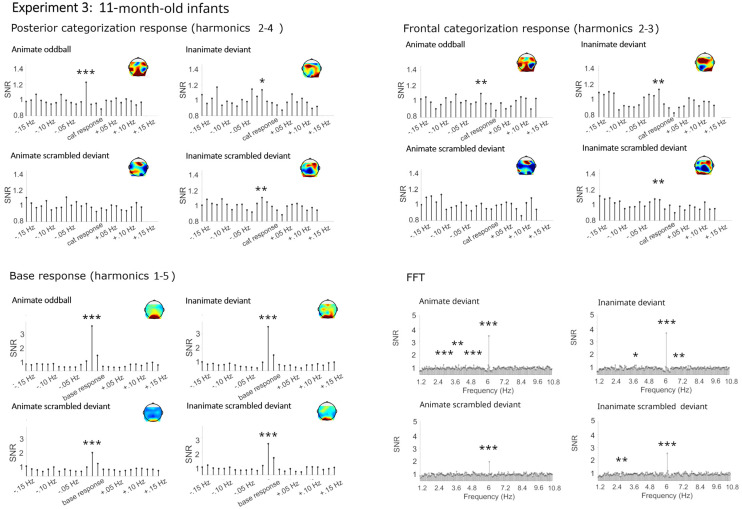
Electroencephalographic responses in Experiment 3 (11-month-old infants). Signal-to-noise ratios (SNRs) of summed responses for the categorization response at the posterior-occipital and frontal leads and of the base response at the occipital channels. Fast Fourier transformation (FFT) responses at the posterior-occipital channels. Data have been averaged across electrodes per cluster and grand-averaged across participants for display. *** = *p* <0.001, ** = *p* < 0.01, * = *p* < 0.05.

Similar to Experiment 1, mainly significant categorization responses were obtained for living and non-living deviants in both the posterior and frontal cluster (see Figure 4, Figure 5 and Figure 6 and Table 3). Here, small significant but strongly reduced categorization responses were also obtained in the phase-scrambled control sequences.

##### Categorization Responses in Posterior-Occipital Cluster (Harmonics 2–4)

Significant categorization responses were obtained when averaging across channels in all conditions except for the animate scrambled deviant (Zs > 2.48; animate scrambled deviant Z = 1.05). A main effect of condition provided moderate evidence for a stronger categorization response in the original rather than the phase-scrambled images, BF_10_ = 9.15, R^2^ = 0.27 (original *M* = 0.32 µV, *SD* = 0.51; scrambled *M* = 0.09 µV, *SD* = 0.44). There was anecdotal evidence against a main effect of age, category, or any interactions, BFs_10_ < 0.40.

##### Categorization Response in Anterior Cluster (Harmonics 2–3)

Significant categorization responses were obtained when averaging across channels only in the original conditions (Zs > 2.20). The Bayesian ANOVA did not provide strong evidence for stronger categorization responses in sequences with original images, BFs_10_ < 1.31 (original *M* = 0.17, *SD* = 0.40; scrambled *M* = 0.03, *SD* = 0.29). In particular, there was no evidence for an effect of condition, BF_10_ = 1.40, and evidence against an effect of age group, BF_10_ = 0.19.

#### 4.2.2. Base Frequency (Harmonics 1–5)

In all conditions, large responses at the base stimulation frequency and its harmonics were observed over the medial occipital cortex (Zs > 27). Visually, there were no differences between conditions. The Bayesian ANOVA also did not provide evidence for any effects, all BFs_10_ < 1.15.

### 4.3. Summary

Four-, seven- and eleven-month-old infants sorted visual stimuli into living and non-living categories. These findings demonstrate sophisticated visual categorization according to animacy from four months onward, with no apparent development throughout the first year of life.

### 4.4. Control Analyses

As the length of sequences needed to be cut short for developmental populations, control analyses employing only the first 20 s of stimulation were run for Experiments 1 and 2. These control analyses confirm the main results and can be found in Supplementary File S4. Moreover, due to the mixed design, fewer participants were tested per cell than in Experiments 1 and 2. Therefore, another control analysis on a random subset of *N* = 10 from Experiments 1 and 2 can be found in Supplementary S5, which yielded results highly similar to the main analysis reported here.

## 5. General Discussion

The current study investigated the categorization of broad living and non-living visual items in adults, children, and infants in a frequency tagging EEG paradigm. Strong responses and similar categorization patterns were observed across age groups. In all age groups, categorization responses emerged at the posterior-occipital and frontal leads, and the response strength was independent of the target category (i.e., comparison of sequences with living or non-living items presented at the target positions). Moreover, significant but distinctly reduced categorization responses were evident in the control sequences with images containing only low-level visual information, pointing to the role of high-level Gestalt information for driving categorization from the earliest age studied here (four months).

Adults and preschool children in this study (Experiments 1, 2) showed similar response patterns compared to a recent study testing animal/furniture categorization [16] including similar areas of activation and severely reduced responses for phase-scrambled sequences. Categorization responses were observed in a posterior and frontal cluster, which suggests that both perceptual and higher-order processes may contribute to categorization. While low-level image characteristics were sufficient to elicit categorization in the phase-scrambled conditions, categorization was markedly enhanced for configurational images containing high-level Gestalt information. This is likely due to the differential power-spectra of living and non-living (man-made) objects [18] that are preserved by phase-scrambling. Together, these studies suggest that little development in broad categorization abilities takes place after children reach school-age. Only the influence of the target category varied between studies: in the precursor study, children showed enhanced responses for animal targets, whereas no difference between the target categories was detected in the present case. We can only speculate about this difference between the two tasks. The living and non-living categories were more diverse than animals and furniture items, and were constructed of six sub-categories each. It may be that the enhanced diversity of the non-living category increased the children’s attention to these items, thus canceling out potential advantages for detecting animals in the visual environment. Future work is needed to test under which circumstances children show an animacy bias.

Crucially, Experiment 3 investigated categorization across the first year of life in three age groups (4, 7, and 11 months). Based on previous work indicative of a global-to-basic-level shift in categorization early in life [4,11], it was expected that the infants would be able to categorize images based on high-level visual cues on such a broad level from an early age. Indeed, high-level categorization was evident in all three infant age groups, and the evidence spoke against developmental changes. Moreover, significant categorization responses were detected in both the posterior-occipital and a frontal cluster, indicating that both visual and higher-level cognitive processes contribute to the living/non-living categorization from four months onward. This is in contrast to the categorically more constrained animal–furniture contrast, where stronger responses for the original compared to phase-scrambled images were not present prior to eleven months of age, and no frontal activation was detected [16]. No differences in categorization strength for living and non-living targets were observed, similar to the older participants (Experiments 1 and 2). In a previous study, an advantage for animals compared to furniture targets was observed at eleven months, but not at younger ages [16]. Global categorization from four months on is compatible with data from looking-time studies [8,23]. Early broad categorization has also been demonstrated in ERP studies [16,30], whereas the signatures of event-related oscillations have so far only been investigated for more circumscribed categories such as houses or faces [19]. Together, these findings strengthen the assumption of a shift in visual categorization from broader to more fine-grained levels in the first year of life, and demonstrate that categorization is based on high-level visual cues from four months onward.

Despite these markers of high-level categorization, the infants’ performance was limited compared to the older participants. While statistical comparisons across age groups were not possible due to differences in the stimulation duration (60 s in adults, 40 s in children, 20 s in infants), qualitative comparisons across experiments indicated less mature categorization in the infants. Effect sizes of high-level categorization were descriptively smaller in the infant participants, and the percentage of infant subjects showing significant categorization responses was more restricted. Moreover, categorization responses were confined to fewer harmonics (2–3 compared to 6–11 in older participants), and no significant response was detected in the first harmonic (1.2 Hz), probably due to a high amount of noise in the low frequency spectrum typical for infant EEG. In contrast, responses to the base stimulation occurred across similar harmonics in all age groups, speaking against general differences in brain responses to frequency tagging associated with age.

To disentangle cognitive from methodological effects, control analyses with sequence durations and participant numbers matched to infant data were performed on the data from the adults and children (Supplementary Files S4 and S5). These demonstrate that differences in the range and number of harmonics remained, even when restricting the available data of the adult and child participants to match the infant data, and significant responses were present in the first harmonic anyway. The major difference compared to the main analysis was that comparatively fewer children showed significant categorization responses when only 20 s of stimulation was analyzed. Based on these control analyses, it seems that differences in elicited categorization harmonics reflect the true differences between age groups, whereas the presence of significant responses in individual averages may be influenced by sequence duration. Of course, it remains a challenge to determine whether other, non-cognitive differences between age groups (such as the presence of movement artefacts) contribute to the distribution of categorization across harmonics.

Employing EEG responses as the dependent variable also allowed us to identify two scalp regions, the occipito-temporal and frontal area, where categorization responses emerged. While source analysis would be necessary to identify the brain regions where these responses were generated, it seems likely that in addition to activations in the visual cortices, a more frontally located region is active. A cortical source analysis of categorization responses would therefore be a promising pathway to further our understanding of developmental trajectories in categorization abilities. An interesting area for future research is whether the frontal activation observed here is related to an activation of cognitive concepts, enabling subjects to form predictions about the prospective behavior of visual objects. Based on findings from behavioral paradigms, such predictions can be expected from at least seven months of age, but earlier ages have not been tested previously [31,32,33]. The present study employed highly similar methods across ages to evaluate categorization patterns across development, a strength compared to behavioral work that is not suited for investigating subjects across the life-span. Moreover, the role of low-level visual cues to categorization was investigated, with evidence showing that low-level cues suffice, but do not fully explain categorization responses. In all ages tested, high-level visual cues including Gestalt information boosted categorization responses. However, the current study was limited by differences in the stimulation duration and analysis of harmonics between age groups (but see the control analyses in Supplementary Files S4 and S5). Moreover, only two conditions were tested within-subject in the infant participants, precluding statistical analyses across experiments, and unfortunately it is not possible to share original data due to limitations of informed consent. Finally, the dropout rates in developmental populations were relatively high, particularly in preschool children due to limits of attentional span and compliance.

## 6. Conclusions

Together, the present study demonstrates a high-level categorization of visual objects into broad classes of living and non-living items in infants, children, and adults. While EEG markers associated with categorization underwent qualitative change from infancy to childhood, the categorization patterns did not change with age, and high-level categorization was present from four months until adulthood. Future work is needed to connect categorization performance to cognitive concepts, and investigate how categorization enables humans across their life-span to make predictions about complex properties and the future behavior of living and non-living objects. It seems likely that proficient early identification of living entities enables humans from infancy to form broad predictions about the future behavior of objects and facilitates successful interactions with living and non-living objects.

### Contributions

1. Neural correlates revealed the categorization of living and non-living visual objects in infants, children, and adults;
2. At all ages, categorization is based on high-level visual cues;
3. Categorization emerges in the posterior-occipital and frontal areas.

## Figures and Tables

**Table 1:** Baseline corrected amplitude (bca) means and standard deviations (SD), Z-score, and signal-to-noise ratio (SNR) ranges for categorization and base rate responses in Experiment 1 (adults). Responses were averaged within electrode clusters. Bca values represent grand-averages across participants and channels, and Z-score and SNR ranges represent individual averages across channels. Z-scores: percentage in parenthesis indicates the portion of participants with a significant response (Z > 1.64).

Response	Condition	Deviant Category	Bca Mean	Bca SD	Z-Score Range	SNR Range
Posterior categorization response (harmonics 1–11)	Original	Animal	0.38	0.12	3.05–16.79 (100%)	1.30–2.34
		Furniture	0.41	0.14	3.17–17.59 (100%)	1.25–2.23
	Phase-scrambled	Animal	0.12	0.08	−0.91–9.51 (70%)	0.94–1.48
		Furniture	0.15	0.08	1.14–8.76 (85%)	1.11–1.68
Anterior categorization response (harmonics 1–8)	Original	Animal	0.23	0.11	3.13–16.32 (100%)	1.11–2.06
		Furniture	0.24	0.13	2.97–17.67 (100%)	1.05–2.10
	Phase-scrambled	Animal	0.07	0.07	−0.28–9.46 (70%)	0.87–1.38
		Furniture	0.10	0.08	0.82–8.61 (85%)	0.97–1.60
Base response (harmonics 1–4)	Original	Animal	1.21	0.43	17.42–51.00 (100%)	3.55–15.92
		Furniture	1.26	0.53	19.24–48.20 (100%)	3.78–17.81
	Phase-scrambled	Animal	1.42	0.78	11.91–50.25 (100%)	3.12–19.01
		Furniture	1.37	0.81	12.96–47.78 (100%)	4.13–18.82

**Table 2:** Baseline corrected amplitude (bca) means and standard deviations (SD), Z-score, and signal-to-noise ratio (SNR) ranges for categorization and base rate responses in Experiment 2 (5–6-year-old children). Responses were averaged within electrode clusters. Bca values represent grand-averages across participants and channels, and Z-score and SNR ranges represent individual averages across channels. Z-scores: percentage in parenthesis indicates the portion of participants with a significant response (Z > 1.64).

Response	Condition	Deviant Category	Bca Mean	Bca SD	Z-Score Range	SNR Range
Posterior categorization response (harmonics 1–12)	Original	Animal	1.63	0.70	2.04–20.52 (100%)	1.19–2.48
		Furniture	1.53	0.63	2.99–14.16 (100%)	1.23–2.34
	Phase-scrambled	Animal	0.47	0.44	−3.28–6.58 (59%)	0.86–1.48
		Furniture	0.36	0.44	−2.97–5.47 (55%)	0.78–1.39
Anterior categorization response (harmonics 1–7)	Original	Animal	0.66	0.51	−0.74–18.94 (81%)	0.95–2.26
		Furniture	0.69	0.52	−1.25–7.28 (77%)	0.86–2.00
	Phase-scrambled	Animal	0.24	0.36	−1.51–11.04 (41%)	0.86–1.58
		Furniture	0.17	0.44	−3.01–6.95 (32%)	0.80–1.59
Base response (harmonics 1–5)	Original	Animal	1.97	0.94	1.65–35.05 (100%)	1.26–7.31
		Furniture	2.12	1.15	2.25–59.05 (100%)	1.58–9.02
	Phase-scrambled	Animal	1.87	1.11	2.96–38.30 (100%)	1.64–7.52
		Furniture	1.85	0.96	1.56–40.62 (95%)	1.24–6.85

**Table 3:** Baseline corrected amplitude (bca) means and standard deviations (SD), Z-score and signal-to-noise ratio (SNR) ranges for categorization and base rate responses in Experiment 3 (4-, 7- and 11-month-old infants). Responses were averaged within electrode clusters. Bca values represent grand-averages across participants and channels, and Z-score and SNR ranges represent individual averages across channels. Z-scores: percentage in parenthesis indicates the portion of participants with a significant response (Z > 1.64).

Response	Condition	Deviant Category	Age Group	N	Bca Mean	Bca SD	Z-Score Range	SNR Range
Posterior-occipital categorization response (harmonics 2–4)	Original	Animal	4	11	0.36	0.69	−1.99–5.54 (18%)	0.80–1.41
			7	9	0.37	0.46	−0.48–3.73 (33%)	0.95–1.45
			11	12	0.52	0.43	−0.74–12.54 (67%)	0.92–1.71
		Furniture	4	13	0.09	0.54	−1.33–2.45 (31%)	0.87–1.33
			7	15	0.30	0.34	−1.28–3.70 (40%)	0.87–1.44
			11	9	0.31	0.58	−1.53–3.95 (33%)	0.86–1.71
	Phase-scrambled	Animal	4	11	0.15	0.49	−0.93–2.94 (27%)	0.90–1.30
			7	9	−0.05	0.46	−3.58–1.78 (11%)	0.73–1.18
			11	12	0.08	0.37	−0.91–2.15 (8%)	0.81–1.28
		Furniture	4	13	0.00	0.43	−1.90–1.56 (0%)	0.76–1.20
			7	15	0.08	0.44	−2.01–3.59 (20%)	0.75–1.41
			11	9	0.31	0.46	−0.71–5.03 (33%)	0.93–1.60
Frontal categorization response (harmonics 2–3)	Original	Animal	4	11	−0.02	0.35	−1.38–4.42 (36%)	0.80–1.63
			7	9	0.16	0.27	−1.41–3.55 (11%)	0.77–1.28
			11	12	0.15	0.33	−1.52–6.63 (25%)	0.64–1.73
		Furniture	4	13	0.10	0.40	−2.47–9.91 (31%)	0.73–1.60
			7	15	0.35	0.50	−1.62–7.24 (33%)	0.93–1.71
			11	9	0.21	0.22	−0.01–3.25 (33%)	1.00–1.60
	Phase-scrambled	Animal	4	11	0.08	0.28	−1.31–2.82 (18%)	0.87–1.39
			7	9	0.01	0.20	−1.50–1.10 (0%)	0.81–1.19
			11	12	0.03	0.32	−2.25–3.43 (8%)	0.72–1.32
		Furniture	4	13	−0.10	0.28	−2.80−0.92 (0%)	0.69–1.18
			7	15	0.07	0.28	−1.45–2.78 (13%)	0.80–1.45
			11	9	0.12	0.37	−1.19–3.93 (11%)	1.05–3.47
Base response (harmonics 1–5)	Original	Animal	4	11	1.56	0.86	3.42–13.21 (100%)	1.62–3.54
			7	9	2.05	1.21	−0.62–49.39 (91%)	0.95–4.83
			11	12	2.91	1.74	3.33–35.61 (100%)	1.80–7.30
		Furniture	4	13	2.54	1.18	4.35–23.25 (100%)	1.72–7.47
			7	15	2.14	1.03	3.08–25.25 (100%)	1.55–6.18
			11	9	2.90	1.09	3.83–30.24 (100%)	1.88–8.09
	Phase-scrambled	Animal	4	11	1.90	0.93	1.25–18.02 (91%)	1.22–4.95
			7	9	1.67	1.53	−0.16–25.16 (67%)	0.93–5.98
			11	12	1.38	1.23	−0.03–19.14 (75%)	0.96–3.95
		Furniture	4	13	3.08	1.52	2.04–25.36 (100%)	0.95–4.83
			7	15	2.02	1.04	1.81–27.03 (100%)	1.49–6.80
			11	9	2.77	2.64	1.20–17.98 (89%)	1.33–6.62

## Data Availability

The data necessary to reproduce the analyses presented here are not publicly accessible as the participants’ informed consent did not include public data sharing, but are available from the first author upon reasonable request.

## Supplementary Materials

The following supporting information can be downloaded at: https://www.mdpi.com/article/10.3390/brainsci14060541/s1, S1: Methods; S2: Processing steps; S3: Analyses including electrode factor; S4: Analyses for 20-s segments; S5: Analyses with a subset of 10 participants.
