# Supplementary material for: The Development of Global-Level Categorization: Frequency Tagging EEG Responses
Source: Brain Sci. 2024 May 24;14(6):541. doi: 10.3390/brainsci14060541 (PMC11201576; doi:10.3390/brainsci14060541)
Supplement: Supplementary file 1 [file brainsci-14-00541-s001.zip › brainsci-2961614-supplementary.pdf]

Supplementary Material

S1 Methods

EEG recordings. The signal was amplified using an amplifier with an input impedance of 10 MΩ and a resolution of .1 μV per bit. Impedances were considered acceptable if < 20 kΩ. Recordings were acquired in a dimly-lit and quiet room.

Stimuli/Presentation. Sixty images each of various living and non-living visual objects were presented. The living category consisted of 10 exemplars each of humans, mammals, birds, insects, reptiliae, and amphibiae. The non-living category contained 10 exemplars each of furniture items, vehicles, buildings, electrical devices, kitchen utensils, and tools. Mean and standard luminance as well as pixel size were equalized across categories. Similar numbers of specific orientations (left, right, centered), expansions (wide, high, square), and colors (brown, gray, colourful) were included for the two categories. To further increase similarity between the two categories, only 50% of living things had visible eyes or a face configuration. Likewise, only 50% of non-living items had a completely straight/edgy outline (others at least partly curvy). All images can be found in supplementary material (S1). For control sequences, phase-scrambled versions of images were created using a custom Matlab script which combines the amplitude spectrum of the original image with the phase of a random white noise image, keeping low-level visual features such as amplitude spectrum and colour contrast constant.

Image background was light grey, and pixel size was approximately 640 (width) x 480 (height; 18 x 13 degrees of visual angle). Stimulation sequences were displayed using a custom Java program, with presentation rate set at 6 Hz using sinusoidal contrast modulation. Each cycle lasted ~166 ms (i.e., 1000 ms/6). Sequences started with a uniform grey background. The stimulation was gradually faded in by progressively increasing the modulation depth from 0% to 100% maximum contrast level (and faded out vice versa). Full image contrast was reached at 83 ms and decreased at the same rate.

The complete image set was presented in a random order before the set was repeated (with the exception that the first image of a block could not be the same as the last image of the previous block). The fade-in was presented for 2 seconds, the main sequence for 60 seconds, followed by a 2-seconds fade-out (overall sequence duration 64 seconds). Stimulus fade-in and fade-out were employed to avoid abrupt eye-movements or blinks. Per sequence, 309 standards and 77 deviants (386 stimuli

in total) were presented. Between sequences, short breaks were provided if needed. Overall, testing took about 35 minutes. At the start of each sequence, triggers were sent to the recording computer via parallel port. Participants were monitored via video and recordings were initiated manually when participants looked attentively at the screen and showed an artifact-free EEG signal.

Full Image Set

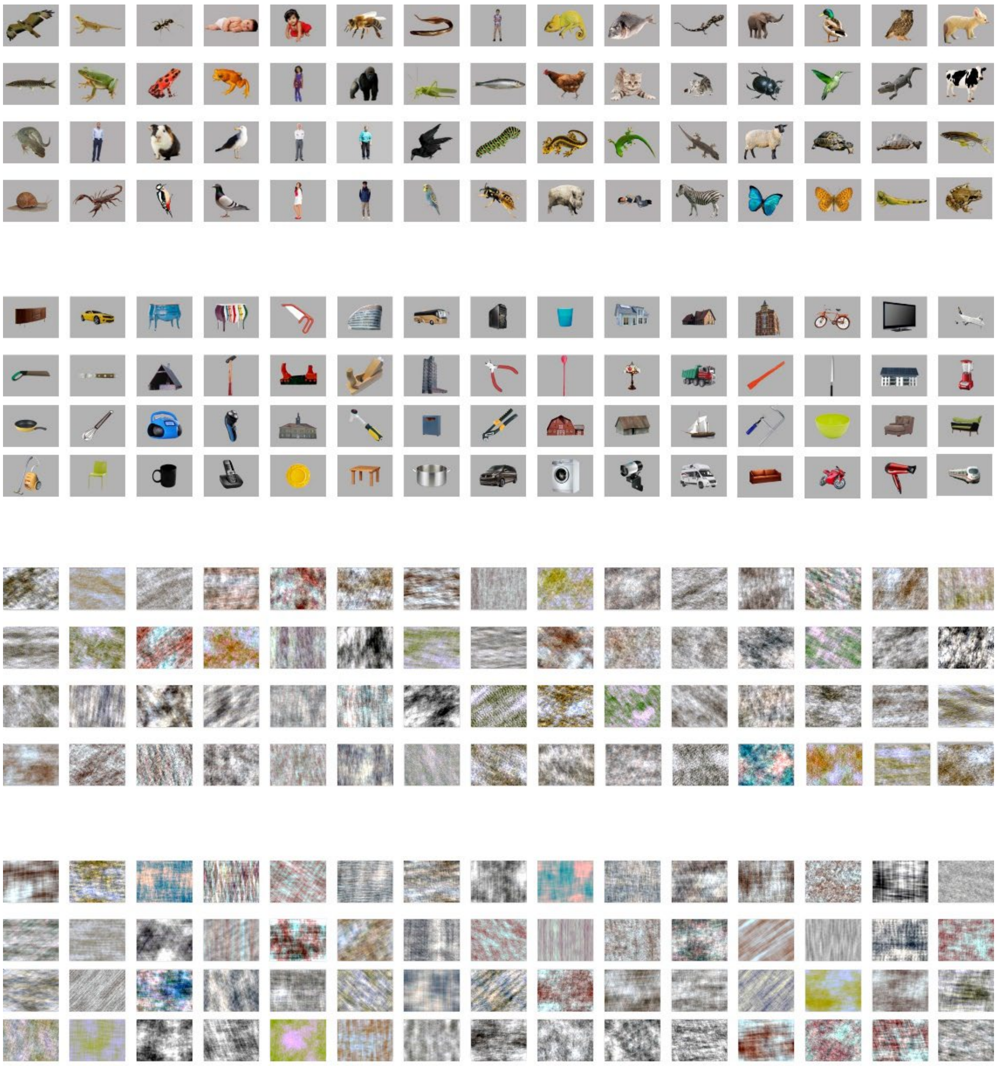

Figure S1. Complete stimulus set: living, non-living, living scrambled, non-living scrambled.

S2 Processing Steps

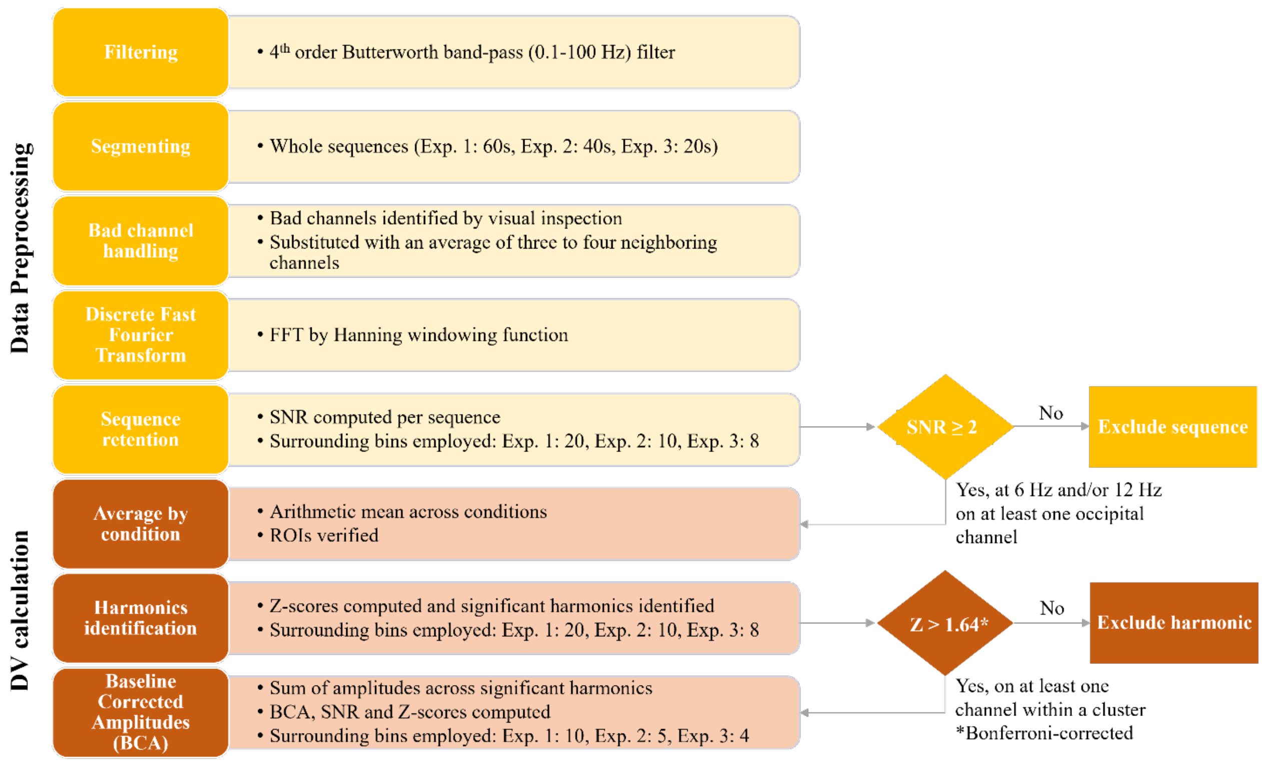

EEG preprocessing. EEG data was band-pass filtered at 0.1-100 Hz using a Butterworth filter with a slope of 24 dB/octet. Data were segmented from 2 seconds before until 2 seconds after the sequence, resulting in 68-second segments in Experiment 1 (-2 s – 66 s), 48-second segments in Experiment 2 (-2 – 46 s), and 28-second segments in Experiment 3 (-2 – 26 s). Next, artifacts were identified based on visual inspection and the following criteria: 1. high amplitudes, 2. amplitude jumps, 3. dead channels, 4. bridged channels. Visual inspection was performed blind to experimental condition. Noisy channels were replaced by interpolating 3-4 surrounding channels per sequence, and a common average reference computation was applied to all channels. If more than 3 (>10%) channels required interpolation, the sequence was discarded.

Frequency domain analyses. Preprocessed data segments were cropped to an integer number of 6 Hz cycles (after fade-in and before fade-out; 15,000 time bins in total in Experiment 1, 10,000 time bins in Experiment 2, and 5,000 time bins in Experiment 3). For each of the four conditions, sequences were averaged in the time-domain for every individual participant, thereby reducing EEG activities non-phase-locked to the stimulus. A discrete Fourier Transformation (DFT) was performed via a fast Fourier

Transformation (FFT) algorithm with a Hanning window (over amplitude, providing only the first half of the spectrum and normalizing amplitude).

The frequency spectrum was sliced into separate chunks centered around each harmonic (i.e., 1.2 Hz, 2.4 Hz….) and then summed. The number of significant harmonics was determined based on Z-scores of grand-averages for original and phase-scrambled conditions (averaged across participants and deviant category), applying Bonferroni-corrections for the number of tests (number of electrodes, depending on the cluster, and conditions, 2). Thus, grand-averages were scanned for harmonics reaching a $Z >= 2.58$ in the posterior-occipital cluster (11 electrodes), $Z >= 2.82$ in the frontal cluster (10 electrodes), and $Z >= 2.4$ for the base response in the occipital cluster (3 electrodes). In Experiment 1, in the posterior-occipital cluster, harmonics 1-11 reached significance and were summed (excluding the base frequency and harmonics; 6 and 12 Hz), and in the frontal cluster, harmonics 1-8 were summed. At the base rate, significant responses were obtained in harmonics 1-4. In Experiment 2, the categorization response was summed for the posterior-occipital cluster of electrodes across harmonics 1-12, at the anterior cluster across harmonics 1-7 (always excluding the base frequency and harmonics), and the base response across harmonics 1-5. In Experiment 3, the categorization response was summed up for the posterior-occipital cluster of electrodes across harmonics 2-4, at the anterior cluster across harmonics 2-3, and the base response across harmonics 1-5.

S3 Analyses Including Electrode Factor

Experiment 1

The within-subjects ANOVAs were composed as condition (2: original, scrambled) * deviant category (2: animal, furniture) * electrode (posterior-occipital categorization response: 11, P3, P4, Pz, P7, P8, PO9, PO10, O1, O2, Oz, Iz; anterior categorization response: 10, F3, F4, Fz, C3, C4, Cz, FC1, FC2, FC5, FC6; base response: 3, O1, O2, Oz).

Categorization responses

As described in the main manuscript, strong and highly significant categorization responses were obtained for living and non-living deviants in both clusters, consistent with rapid categorization in visual and anterior networks. Small but significant categorization responses were also obtained for phase-scrambled control sequences.

Categorization responses in posterior-occipital cluster (harmonics 1-11). A main effect of condition provided extreme evidence for a stronger categorization response in original than phase-scrambled images, BF_{10} = 7.85e^{13}, R^2 = .34 (original *M* = .76, SD = .3; scrambled *M* = .28, SD = .1). Moreover, there was extreme evidence for an effect of electrode, BF_{10} = 7.85e^{13}, R^2 = .34. Post-hoc tests revealed that strongest responses were obtained on occipital channels, which did not differ strongly from one another (BFs_{10} > .17 < 3.07), but from all other channels (BFs_{10} > 8.63) except for P8 which did not differ strongly from O2 and Oz (BFs_{10} < 2.46). Evidence spoke against other main effects or interactions, all BFs_{10} < .25. In particular, there was strong evidence against a main effect of category, BF_{10} < .25, or an interaction of category and condition, BF_{10} < .09. A significant categorization response was obtained in all participants and conditions (averaged across channels).

Categorization response in anterior cluster (harmonics 1-8). There was extreme evidence for effects of condition, BFs_{10} = 8.94 e^{13}, and electrode, BFs_{10} > 51,710. Stronger responses were observed for original than phase-scrambled images (original *M* = .46, SD = .2; scrambled *M* = .14, SD = .1). Post-hoc tests revealed that strongest responses were obtained on fronto-central leads (FC1, FC2, Cz, Fz), which did not differ from each other (BFs_{10} < .18), but from all other channels (BFs_{10} > .13 < 5,927). An interaction of condition and electrode, BF_{10} > 3.26 was followed up by comparing conditions separately per electrode. At all electrodes, decisive evidence for stronger responses in original sequences was observed, but with variable Bayes

Factors (smallest difference at C4, BF = 160.53; strongest difference at FC1, BF = $1.58e^7$).

Base frequency (harmonics 1-4).

Large responses at the base stimulation frequency and its harmonics were observed in all conditions over the medial occipital cortex, with a maximum at O1, O2, Oz. Evidence spoke against any differences between conditions or electrodes were observed, BFs < .12.

Summary.

Together, supplemental analyses including the factor electrode are consistent with the main analysis, showing that adults quickly and expertly categorize stimuli based on animacy, and that this ability is partly based on low-level cues. No interaction with condition or category emerged, indicating that responses were strongest at occipital and fronto-central leads regardless of images presented.

Experiment 2

The within-subjects ANOVAs were composed as condition (2: original, scrambled) * deviant category (2: animal, furniture) * electrode (posterior-occipital categorization response: 11, P3, P4, Pz, P7, P8, PO9, PO10, O1, O2, Oz, Iz; anterior categorization response: 10, F3, F4, Fz, C3, C4, Cz, FC1, FC2, FC5, FC6; base response: 3, O1, O2, Oz).

Categorization Responses

Similar to Experiment 1, strong and highly significant categorization responses were obtained for living and non-living deviants in both the posterior and the frontal cluster. Small significant but strongly reduced categorization responses were obtained in phase-scrambled control sequences.

Categorization Response in Posterior-Occipital Cluster (harmonics 1-12). There was extreme evidence (BFs_{10} > 84,393) for effects of condition, electrode, and a condition by electrode interaction. Stronger responses were observed for original than phase-scrambled images, $BF_{10} = 3.70e^{13}$. The effect of electrode ($BF_{10} = 3.70e^{13}$, $R^2 = .48$) reflected stronger responses at O1, O2 and Oz compared to the other electrodes (Os *M* = 1.34, SD = .7, other electrodes *M* = .70, SD = .6; BFs within O cluster < 2.22, BFs contrasting Os and other electrodes > 25, except for PO10, which did not differ from O1, $BF_{10} = 2.56$). Moreover, strong responses were registered at Iz, PO9 and PO10 (stronger than P3, P4, Pz, all $BFs_{10} > 10$). The

interaction between condition and electrode, $BF_{10} > 84,393$, reflected variability in the strength of condition effects per electrode. Strong evidence ($BFs_{10} > 19$) for condition effects was obtained on all electrodes except P3 ($BF_{10} = 1.30$). Evidence spoke against any other interactions, all $BFs_{10} < .12$.

Categorization response in anterior cluster (harmonics 1-7). There was extreme evidence ($BF_{10} = \infty$) for an effect of condition, and evidence against any other effects, all $BFs_{10} < .04$.

Base Response (harmonics 1-5). In all conditions, large responses at the base stimulation frequency and its harmonics were observed over the medial occipital cortex. There was moderate evidence for a main effect of electrode, $BF_{10} = 3.04$, with stronger responses at Oz (O1 $M = 1.81$, SD = 1.08, O2 $M = 1.83$, SD = .81, Oz $M = 2.21$, SD = 1.12), $R^2 = .59$. Evidence spoke against any other main effects or interactions, all $BFs_{10} <= .24$.

Summary.

Together, supplemental analyses including the factor electrode are consistent with the main analysis. 5-6-year-olds, like adults, quickly and expertly categorize stimuli based on animacy, partly reflecting processing of low-level cues. Categorization responses were strongest at occipital leads, and stronger responses for original images were observed on all electrodes except for P3.

Experiment 3

The mixed ANOVAs were composed as condition (within-subjects factor, 2: original, scrambled) * deviant category (between-subjects factor, 2: animal, furniture) * electrode (within-subjects factor, posterior-occipital categorization response: 11, P3, P4, Pz, P7, P8, PO9, PO10, O1, O2, Oz, Iz; anterior categorization response: 10, F3, F4, Fz, C3, C4, Cz, FC1, FC2, FC5, FC6; base response: 3, O1, O2, Oz).

Categorization Responses

Similar to Experiment 1, mainly significant categorization responses were obtained for living and non-living deviants in both the posterior and the frontal cluster. Here as well, small significant but strongly reduced categorization responses were obtained in phase-scrambled control sequences.

Categorization Response in the Posterior-Occipital Cluster (harmonics 2-4). There was very strong evidence for a main effect of condition, $BF_{10} = 72.80$,

consistent with the main analysis, and demonstrating stronger responses for original images. Evidence spoke against any other effects, $BFs_{10} < .10$, and in particular against an effect of electrode, $BF_{10} = 5.92e^{-4}$.

Categorization response in anterior cluster (harmonics 2-3). Evidence spoke against any main effects or interactions, $BFs_{10} <= .86$, particularly against an effect of electrode, $BF_{10} = 3.25e^{-5}$.

Base Response (harmonics 1-5). In all conditions, large responses at the base stimulation frequency and its harmonics were observed over the medial occipital cortex. There was a main effect of electrode, $BF_{10} > 3,769.39$, $R^2 = .63$. Post-hoc tests indicated stronger responses on Oz ($BFs_{10} > 16,8383$) than O1 and O2, which did not differ from each other ($BF_{10} = .16$). In addition, there was evidence for main effects of condition, $BF_{10} = 41.90$, $R^2 = .38$, and an interaction between condition and age-group, $BF_{10} = 99.53$, $R^2 = .40$. When looking separately at the three age-groups, electrode effects emerged only at 4 and 7 months ($BFs_{10} > 6.97$), whereas the condition effect was present only at 11 months, $BF_{10} = 18.69$. There was no evidence for any other main effects or interactions, all $BFs_{10} < 2.58$.

Summary.

Consistent with the main analysis, four-, seven- and eleven-month-old infants sorted visual stimuli into living and non-living categories based on high-level visual cues. No electrode effects emerged for the categorization response, whereas the base response was strongest at Oz at four and seven months of age.

S4 Analyses for 20-second-segments

Experiment 1

To ensure that differences between age-groups did not result from shorter sequences in infant participants, a control analysis using only 20 second segments (5,000 time bins) of the adult data (discarding the last 40 seconds of each sequence) was run. Similar to the main analyses, significant harmonics were identified based on Z-scores of grand-averaged data for original and phase-scrambled conditions (averaged across participants and deviant category), applying Bonferroni-corrections for the number of tests (number of electrodes, depending on the cluster, and conditions, 2). In the occipital-posterior cluster, categorization harmonics 1-11 reached significance, and in the frontal cluster, harmonics 1-7. For the base rate, harmonics 1-4 reached significance and were summed.

Categorization responses

Conforming to the main analysis, strong categorization responses were observed across harmonics in the posterior-occipital and the frontal cluster. Categorization was also observed but considerably reduced for phase-scrambled images.

Table S1. Baseline corrected amplitude (bca) means and standard deviations (*SD*), Z-score and signal-to-noise ratio (SNR) ranges for categorization and base rate responses averaged within electrode clusters in 20-second-segments of Experiment 1 (adults). Bca values represent grand-averages across participants and channels, and Z-score and SNR ranges represent individual averages across channels. Z-scores: Percentage in parenthesis indicate the portion of participants with a significant response (Z > 1.64).

Response	Condition	Deviant category	Bca mean	Bca SD	Z-score range	SNR range
Posterior categorization response (harmonics 1-11)	Original	Animal	.31	.15	.69-10.48 (80%)	1.07-1.99
		Furniture	.24	.11	.91-10.91 (85%)	1.05-1.54
	Phase-scrambled	Animal	.07	.09	-1.21-2.88 (35%)	.87-1.22

		Furniture	.07	.11	-1.46-7.24 (20%)	.89-1.41
Anterior categorization response (harmonics 1-7)	Original	Animal	.16	.14	-.56-6.36 (55%)	.94-1.71
		Furniture	.14	.10	-.47-4.86 (50%)	.98-1.45
	Phase-scrambled	Animal	.01	.10	-3.08-4.31 (10%)	.76-1.45
		Furniture	.03	.13	-1.36-4.82 (25%)	.75-1.45
Base response (harmonics 1-5)	Original	Animal	1.00	.42	6.53-52.64 (100%)	1.94-9.25
		Furniture	1.00	.44	5.84-37.31 (100%)	2.11-9.29
	Phase-scrambled	Animal	1.19	.73	4.91-45.19 (100%)	1.83-11.59
		Furniture	1.12	.71	4.62-47.24 (100%)	2.16-10.58

Categorization responses in posterior-occipital cluster (harmonics 1-11). Significant categorization responses were obtained when averaging across channels in all four conditions (all Zs > 2.48). A main effect of condition provided extreme evidence for a stronger categorization response in original than phase-scrambled images, $BF_{10} = 5.99e^9$, $R^2 = .73$ (original $M = .27$, SD = .10; scrambled $M = .07$, SD = .083). In particular, there was evidence against a main effect of category, or an interaction of category and condition, $BFs_{10} < .98$.

Categorization response in anterior cluster (harmonics 1-7). Significant categorization responses were obtained when averaging across channels in all conditions (Zs > 1.87) except furniture deviant scrambled (Z = 1.60). There was conclusive evidence for stronger responses in original than phase-scrambled images, $BF_{10} > 63.597$, $R^2 = .42$ (original $M = .15$, SD = .08; scrambled $M = .02$, SD = .10). Evidence spoke against any other effects, $BF_{10} < .81$.

Base frequency (harmonics 1-4).

Large responses at the base stimulation frequency and its harmonics were observed in all conditions over the medial occipital cortex, with a maximum at O1, O2, Oz. All responses were significant (Zs > 16). Evidence spoke against any main effects or interactions, all $BFs_{10} < .95$.

Summary.

Supplemental analyses employing 20-second-sequences of data obtained with adult participants largely conform to the main analysis, showing that adults quickly and expertly categorize stimuli based on animacy.

Experiment 2

To ensure that differences between age-groups did not result from shorter sequences in infant participants, a control analysis using only 20 second segments (5,000 time bins) of children's data (discarding the last 20 seconds of each sequence) was run. For this analysis, 2 additional participants were excluded as they did not have trials left showing a sufficient base rate response (SNR > 2), leaving a sample of $N = 20$. For the categorization response, in the frontal cluster, harmonics 1-6 reached significance, whereas harmonics 1-7 were significant in the posterior-occipital cluster. For the base rate, harmonics 1-5 reached significance and were summed.

Categorization responses

Similar to Experiment 1, large categorization responses were observed across harmonics in the posterior-occipital and the frontal cluster. Categorization was also observed but severely reduced for phase-scrambled images.

Table S3. Baseline corrected amplitude (bca) means and standard deviations (*SD*), Z-score and signal-to-noise ratio (SNR) ranges for categorization and base rate responses averaged within electrode clusters in Experiment 2 (five-to-six-year-old children). Bca values represent grand-averages across participants and channels, and Z-score and SNR ranges represent individual averages across channels. Z-score values in parenthesis indicate the percentage of participants with a significant response (Z > 1.64).

Response	Condition	Deviant category	Bca mean	Bca SD	Z-score range	SNR range

Posterior categorization response (harmonics 1-7)	Original	Animal	.96	.60	-1.73-2.49 (20%)	.91-1.88
		Furniture	.73	.66	-1.41-3.64 (20%)	.87-1.96
	Phase-scrambled	Animal	.30	.72	-1.47-6.96 (10%)	.85-1.78
		Furniture	.15	.75	-2.18-2.43 (5%)	.70-1.58
Anterior categorization response (harmonics 1-6)	Original	Animal	.36	.96	-3.16-6.36 (15%)	.69-2.02
		Furniture	.63	.90	-2.27-2.40 (15%)	.70-1.77
	Phase-scrambled	Animal	.23	.82	-2.29-7.94 (5%)	.68-1.58
		Furniture	-.03	.72	-2.94-3.13 (10%)	.63-1.46
Base response (harmonics 1-6)	Original	Animal	.67	.43	.80-31.15 (95%)	1.25-7.54
		Furniture	1.08	.48	2.39-31.77 (100%)	1.56-6.55
	Phase-scrambled	Animal	.83	.57	1.15-27.61 (95%)	1.33-4.96
		Furniture	.75	.48	.60-16.96 (85%)	.99-4.94

Categorization response in posterior-occipital cluster (harmonics 1-7). Significant categorization responses were obtained when averaging across channels in all conditions except inanimate scrambled deviant (Zs > 3.90, furniture scrambled deviant Z = 1.52). Stronger responses were obtained in original than phase-scrambled sequences, BF_{10} = 562.00, R^2 = .48 (original *M* = .84, SD = .50; scrambled *M* = .22, SD = .50).

Categorization response in anterior cluster (harmonics 1-6). Significant categorization responses were obtained when averaging across channels in original

conditions ($Zs > 3.76$), but not for scrambled deviants. There was no evidence for an effect of condition ($BF_{10} = 1.79$) or any other effects ($BFs_{10} < .41$).

Base Response (harmonics 1-5)

Large, significant responses at the base stimulation frequency and its harmonics were observed in all conditions over the medial occipital cortex, with a maximum at O1, O2, Oz (all $Zs > 17$). Evidence spoke against any effects, all $BFs_{10} < .32$. In particular, responses did not differ between original and phase-scrambled conditions, $BF_{10} = .22$.

Summary.

Supplemental analyses on 20-second-segments of children's data are consistent with the main analysis, showing that children quickly and expertly categorize stimuli based on animacy, partly reflecting low-level cues. However, no significant responses emerged for scrambled images in the anterior region, revealing that children's categorization response in these conditions are somewhat smaller and require enhanced sequence length for detection.

S5 Analyses with a Subset of 10 Participants

Experiment 1

To ensure that differences between Experiments (that is, age-groups) were not an effect of differences in the number of subjects, a control analysis on a randomly drawn subset of 10 participants was run. In the occipital-posterior cluster, categorization harmonics 1-12 reached significance, and in the frontal cluster, harmonics 1-9. For the base rate, harmonics 1-4 reached significance and were summed.

Categorization responses

Conforming to the main analysis, strong categorization responses were observed across harmonics in the posterior-occipital and the frontal cluster. Categorization was also observed but considerably reduced for phase-scrambled images.

Categorization responses in posterior-occipital cluster (harmonics 1-12). A main effect of condition provided extreme evidence for a stronger categorization response in original than phase-scrambled images, $BF_{10} = 5.33e^{8}$, $R^2 = .85$ (original $M = .41$, SD = .10; scrambled $M = .15$, SD = .06). Evidence spoke against any other effects, $BFs_{10} < .56$.

Categorization response in anterior cluster (harmonics 1-14). There was extreme evidence for stronger responses in original than phase-scrambled images, $BF_{10} = 3.48e^{6}$, $R^2 = .79$ (original $M = .25$, SD = .07; scrambled $M = .09$, SD = .06). Evidence spoke against any other main effect or interaction, $BFs_{10} < .43$.

Base frequency (harmonics 1-7).

Large responses at the base stimulation frequency and its harmonics were observed in all conditions over the medial occipital cortex, with a maximum at O1, O2, Oz. There was no evidence for an effect of condition, $BF_{10} = 1.25$, or any other main effects or interactions, $BFs_{10} < .29$.

Summary. These supplemental analyses on a subset of $N = 10$ participants provide converging evidence that adults quickly and expertly categorize images based on animacy, with no apparent differences compared to the main analysis.

Experiment 2

A control analysis using a subset of 10 participants was conducted. In the occipital-posterior cluster, categorization harmonics 1-16 reached significance, and in the frontal cluster, harmonics 1-7. For the base rate, harmonics 1-6 reached significance and were summed.

Categorization responses

Similar to Experiment 1, large categorization responses were observed across harmonics in the posterior-occipital and the frontal cluster. Categorization was also observed but severely reduced for phase-scrambled images.

Categorization response in posterior-occipital cluster (harmonics 1-16). Significant categorization responses were observed in all conditions (Zs > 6). Responses were stronger in original compared to phase-scrambled sequences, BF_{10} = 1.53e^7, R^2 = .70 (original *M* = 1.68, SD = .62; scrambled *M* = .45, SD = .17), with evidence against any other effects, BFs < .64.

Categorization response in anterior cluster (harmonics 1-7). Significant categorization responses were observed in all conditions (Zs > 2.4). Stronger responses were obtained for original than phase-scrambled sequences, BF_{10} > 15,513, R^2 = .61 (original *M* = .72, SD = .38; scrambled *M* = .16, SD = .22). There was moderate evidence against any other effects, BFs < .24.

Base Response (harmonics 1-6)

Large responses at the base stimulation frequency and its harmonics were observed in all conditions over the medial occipital cortex. Evidence spoke against any effects, all BFs_{10} < .24. In particular, responses did not differ between original and phase-scrambled conditions, BF_{10} = .23.

Summary. The supplemental analyses on a subset of *N* = 10 child participants provide converging evidence for fast and expert categorization based on animacy, with no apparent differences compared to the main analysis.
